# Laparoscopic Repair of a Large Paraesophageal Hernia with Migration of the Stomach into the Mediastinum Creating an Upside-Down Stomach

**DOI:** 10.1155/2017/7428195

**Published:** 2017-07-10

**Authors:** Nasser Sakran, Hadar Nevo, Ron Dar, Asnat Raziel, Dan Hershko

**Affiliations:** ^1^Emek Medical Center, Surgery A, Afula, Israel; ^2^Faculty of Medicine, Technion-Israel Institute of Technology, Haifa, Israel; ^3^Assia Medical Group, Assuta Medical Center, Tel Aviv, Israel

## Abstract

Upside-down stomach is a relatively rare type of a large paraesophageal hernia characterized by the migration of the stomach into the posterior mediastinum. Upside-down stomach is prone to severe complications and therefore surgery is recommended even in asymptomatic patients. A 62-year-old male presented with frequent abdominal pain with nausea and vomiting that persisted for one year. The patient was obese with fatty liver and was treated medically for gastroesophageal reflux disease (GERD) for 4 years. On upper gastrointestinal CT study a level-IV paraesophageal hernia was detected with upside-down stomach, and he was referred for elective surgery. Laparoscopic surgery included reduction of the stomach into the abdominal cavity followed by dissection of the paraesophageal membrane and hernia sac. The hiatal defect was closed using a wound closure device and nonabsorbable sutures. The defect closure was reinforced using Physiomesh tucked anteriorly and sutured posteriorly to the diaphragm. Follow-up was uneventful and the patient is free of complaints. The results of this surgical intervention support previous reports that laparoscopic repair with the use of biological mesh in the setting of large paraesophageal hernia should be favorably considered.

## 1. Introduction

Upside-down stomach (UDS) is an uncommon type of large paraesophageal hernia which is characterized by migration of the stomach into the posterior mediastinum. Although this type of hernia represents a very small percentage of all cases, it has significant clinical importance due to the high risk of development of life threatening complications. As such, surgery for UDS repair is advocated even in asymptomatic patients, since this type of hernia may often present acutely with severe complications such as volvulus, strangulation, rupture, or gangrene of intrathoracic stomach.

## 2. Case Presentation

A 62-year-old male was referred for surgical consultation due to complaints of recurrent abdominal pain accompanied by nausea and vomiting for the past year. The patient received proton pump inhibitors (PPIs) for treatment of reflux symptoms for over 4 years. In addition the patient was obese (Body Mass Index 33.3 kg/m^2^) with known hyperlipidemia and fatty liver.

**Figure 1:**
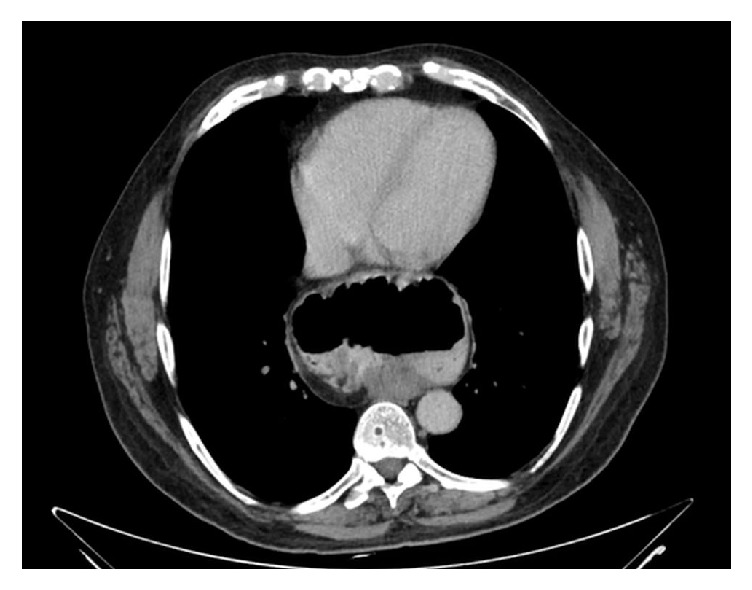
CT scan showing huge hiatal hernia and upside-down stomach.

**Figure 2:**
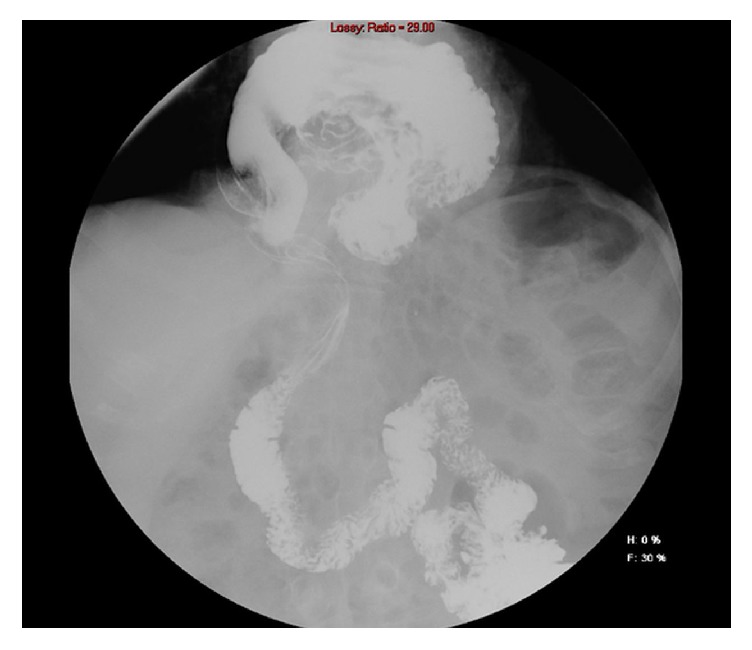
UGIS showing paraesophageal hiatal hernia with upside-down stomach.

Computed tomography (CT) scan and upper gastrointestinal series (UGIS) showed a huge paraesophageal hiatal hernia (level IV) with USD (Figures 1 and 2). Gastroscopy did not reveal mucosal pathology. The full procedure could not be completed since the scope could not be forwarded into the duodenum, suggesting the presence of gastric volvulus.

**Figure 3:**
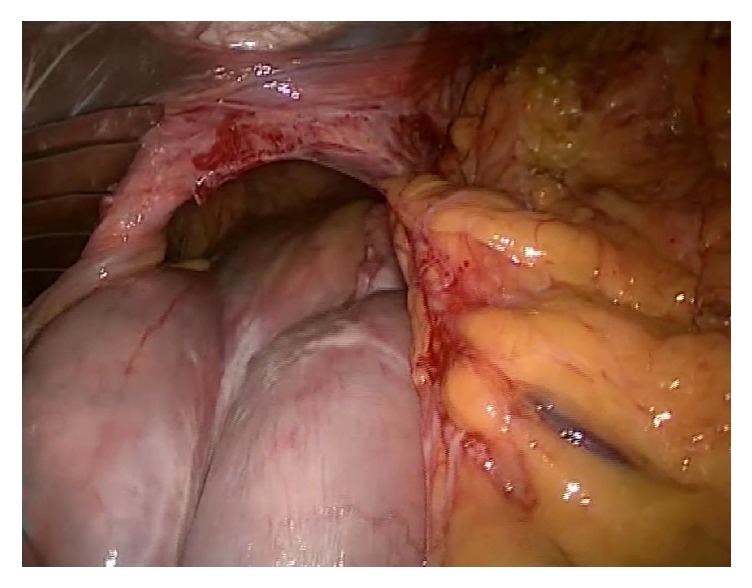
Endoscopic image of abdomen reduction into the abdominal cavity.

The procedure was performed laparoscopically based on Nissen fundoplication. The first step was to reduce the stomach into the abdominal cavity (Figure 3). Following reduction of the stomach, the phrenoesophageal membrane was dissected and the huge hernia sac was revealed and resected. After preparation of the diaphragmatic crus and the distal esophagus, the hiatal defect was closed using V-Loc™ Wound Closure Device, nonabsorbable sutures (Covidien). A tension-free closure of the defect was performed using 10 × 15 cm ETHICON PHYSIOMESH™ Composite Mesh tucked anteriorly and sutured posteriorly to the diaphragm.

The postoperative course was uneventful; the patient was put on liquid diet and was discharged from the department four days after surgery for ambulatory follow-up. He was prescribed with PPIs for 6 weeks and pain relievers. During 3 years' outpatient follow-up the patient is feeling well and is free of complaints.

## 3. Discussion

Hiatal hernia is a condition in which intra-abdominal organs herniate into the thoracic cavity through the esophageal hiatus. UDS is the rarest type of a large paraesophageal hernia with volvulus (<5%) [1], in which there is migration of the stomach into the posterior mediastinum. It is characterized by rotation of the stomach toward the right pleural cavity along the organoaxial axis (defined by the phrenoesophageal membrane at the hiatus and the retroperitoneal attachment of the first portion of the duodenum) [2].

Mortality rates for emergency repair have been reported to be as low as 0–5.4% [3, 4], though average mortality rates for emergency hiatal hernia surgery are around 17% [3]. This high rate has led to the consent that elective surgery to repair UDS is recommended even in asymptomatic patients. Surgical intervention requires reduction of the stomach to the gastric lodge and calibration of the hiatus with the addition of antireflux procedure.

Primary sutured crural repair has been the mainstay of practice for many years, but objective follow-up has suggested very high recurrence rates (42% and higher) after paraesophageal hernia repair [5, 6]. Laparoscopic operation of paraesophageal hernia was first described by Cuschieri in 1992 [7]. Since then, the use of laparoscopic approach for repair of UDS has increased and reports have shown high success rates, lower postoperative morbidity, and shorter hospital stay compared to conventional laparotomy or thoracotomy [8]. However, it was found that the rate of recurrence was significantly higher when the repair was preformed laparoscopically, ranging from 8% to 27% [9]. In most cases, the recurrences of hernias remain asymptomatic and are diagnosed on barium study [10]. Nevertheless, this potential complication must be of concern, and techniques to reduce the recurrence are necessary.

This has prompted many authors to advocate crural repair reinforcement. Indeed, the use of mesh for reinforcement leads to decrease in short term recurrence rates. Most reinforced repairs use some form of mesh. Mesh insertion at the hiatus is suggested as a mean that may decrease the rate of recurrence [11]. Most commonly the mesh is applied in onlay fashion after primary crural closure [12, 13].

The meshes have been fixed by using a variety of different techniques, including various glues, tacks, and sutures. Care should be taken that fixation methods (particularly tacks) do not breach the aorta or pericardium when applied low on the left crus or near the apex of the crura anteriorly.

The need for mesh is an issue of great debate as is the type of mesh (biological versus synthetic).

Complications related to the mesh such as esophageal erosion, ulceration, stricture, dysphagia, pericardial tamponade, and effusion [14, 15] were reported and must be taken into consideration. We decided to use Physiomesh in this particular patient because of the wide hiatal defect.

In conclusion, this minimally invasive technique should be strongly considered in patients with upside-down stomach with expected safety and favorable outcomes.
